# Agricultural soils as a potential reservoir of mycotoxigenic *Fusarium* spp. in northwest Spain

**DOI:** 10.3389/ffunb.2026.1946828

**Published:** 2026-10-06

**Authors:** Rocio Montes-Ruiz, Mario Masiello, Lucia Gambacorta, Annalisa De Girolamo, Natalia Martinez-Reyes, Sara Mayo-Prieto, Antonio Moretti, Pedro A. Casquero, Santiago Gutiérrez

**Affiliations:** 1 Grupo Universitario de Investigación en Ingeniería y Agricultura Sostenible (GUIIAS), Instituto de Medio Ambiente y Cambio Global (IMACG), Universidad de León, León, Spain; 2 Institute of Sciences of Food Production (ISPA), National Research Council (CNR), Bari, Italy

**Keywords:** beauvericin, emerging mycotoxins, enniatins, mycotoxin profiles, phytopathogenic fungi, species complex

## Abstract

*Fusarium* species, which are widely distributed across agricultural ecosystems worldwide, are among the most economically harmful phytopathogens. They can colonize a broad range of agri-food crops, causing plant diseases and the accumulation of mycotoxins in edible plant tissues. *Fusarium* species produce a variety of secondary metabolites, posing toxicological risks to human and animal health. A total of 119 *Fusarium* strains isolated from agricultural soils in northwestern Iberian Peninsula were molecularly identified at the species level, and their capability to produce 17 mycotoxins was evaluated *in vitro* using liquid chromatography coupled to high-resolution mass spectrometry (LC-HRMS). A high *Fusarium* biodiversity was observed, as 15 *Fusarium* species in eight species complexes were detected, some of them not extensively described in Spanish soils, including *Fusarium algeriense*, *Fusarium brachygibbosum*, *Fusarium clavum*, and *Fusarium flagelliforme*. Each *Fusarium* strain showed a specific mycotoxin profile with varying concentrations. Beauvericin and enniatins were the most prevalent at very high levels, and for a number of species, production has not been previously reported. Trichothecenes and fumonisins were less frequently detected than expected from previous reports. The multi-mycotoxin profiles were shaped by species identity, the only significant factor, rather than by soil environmental characteristics. These findings highlight soil as a reservoir of phytopathogenic and mycotoxigenic *Fusarium* spp., contribute to characterizing their toxigenic profiles, and emphasize the need to investigate emerging mycotoxins.

## 1 Introduction

*Fusarium* Link is a genus of filamentous fungi (Ascomycota; Sordariomycetes; Hypocreales) ubiquitous in agronomic ecosystems around the world (Summerell, 2019). The species of this genus are among the most destructive fungal pathogens due to their broad host range and their ability to cause a wide variety of diseases that affect different plant organs and developmental stages, particularly in cereal crops (Ekwomadu and Mwanza, 2023). They also produce a vast amount of secondary metabolites, including polyketides (fusarins, fumonisins, and fusaric acid), terpenes (T-2 toxin, deoxynivalenol, and other trichothecenes), non-ribosomal peptides (enniatins and beauvericins), and other compounds (Desjardins and Proctor, 2007). These metabolites play a role in triggering infection symptoms in plants and pose significant risks to human and animal health; therefore, they are considered toxins. A number of these mycotoxins are especially relevant and well characterized, and their maximum levels are subject to regulatory limits by different organizations for mycotoxins: deoxynivalenol (DON), T-2 toxin (T-2), HT-2 toxin (HT-2), zearalenone (ZEA), and fumonisins (FBs) (Vitalini et al., 2025). Other *Fusarium* mycotoxins are neither routinely determined nor regulated due to limited data on their toxicity and occurrence, although concerns regarding their effect on animal health and the environment are increasing. These are referred to as “emerging mycotoxins,” such as moniliformin, enniatins (ENNs), beauvericin (BEA), and fusaproliferin, and some studies have shown their co-occurrence with “traditional” ones (Jajić et al., 2019). Multi-mycotoxin contamination is frequently observed, both due to the ability of mycotoxigenic fungi to produce more than one metabolite and to the presence of complex fungal communities with different biosynthetic activities (Streit et al., 2012; Han et al., 2014; Stanciu et al., 2017).

*Fusarium* members are often considered soilborne phytopathogens as they can survive in soil and plant debris for long periods, with an especially high diversity in agricultural soils (Daou et al., 2021). Abiotic factors play a critical role in modulating the distribution and pathogenic activity of *Fusarium*, as the pH, organic matter (OM) content, and texture indirectly affect the water holding capacity, nutrient availability, gas exchange, and root growth, which are related to soil suppressiveness (Nikitin et al., 2023). Disease suppressiveness is a characteristic provided by certain microbial soil communities, which is normally enhanced by a higher OM content and a neutral pH (Siegel-Hertz et al., 2018; Deng et al., 2021). Acidic soils (pH 4.5–5.5) have been shown to favor *Fusarium oxysporum*, *Fusarium pseudograminearum*, and *Fusarium culmorum* spore germination and seed infection (Nikitin et al., 2023), while the severity of *Fusarium* wilt of banana caused by *F. oxysporum* f. sp. *cubense* has been observed to increase in both acidic and slightly alkaline soils (pH < 7) by Rodríguez-Yzquierdo et al. (2023) due to the microbial diversity being restricted at low and high pH levels. In addition, OM has been shown to shape the *Fusarium* community composition and richness (Leblanc et al., 2017). Other environmental conditions including high temperature and humidity increase the risk of fungal growth and mycotoxin production, with climate change also being a determining factor likely to aggravate mycotoxin contamination concerns in Mediterranean areas (Daou et al., 2021).

Nonetheless, the effects of these environmental variables are highly dependent on species identity, as genetic features determine the potential for toxin production (Munkvold et al., 2021). Apart from species identity, there are also changes at the strain level due to mutation, recombination, differential gene expression, or horizontal gene transfer (Fitzpatrick, 2012; Kelly and Ward, 2018; Fulcher et al., 2019; Liu et al., 2019; Villani et al., 2019; Beccari et al., 2022), which influence pathogen–host specificity and interactions (Li et al., 2020; Martínez-Soto et al., 2023).

In the present study, we analyzed a collection of *Fusarium* isolates from a variety of agricultural soils in the northwestern Iberian Peninsula to assess their occurrence and mycotoxin production profiles. The isolates were molecularly identified, and the *in vitro* production of 17 toxins on rice cultures was quantified using liquid chromatography coupled to high-resolution mass spectrometry (LC-HRMS). Evaluation of individual strains rather than bulk soil mixture allowed us to directly isolate the occurrence of *Fusarium* mycotoxins and link their production profiles to species identity and complexes and to environmental variables. Overall, these findings clarify the potential risk of the soil as a phytopathogen and a mycotoxin reservoir, improving our understanding of *Fusarium* toxigenic profiles and potential mycotoxin accumulation in crop environments.

## 2 Materials and methods

### 2.1 Soil samples and sampling method

A total of 66 soil samples were collected from different crop fields located in northwestern Spain. Their distribution by province and crop type is summarized in **Table 1**. Sampling was performed at depths of 5–20 cm, as described by Mayo-Prieto et al. (2021), and samples were stored at 4°C until further analyses.

**Table 1:** Distribution of the soil samples by province and crop type.

Province	Vineyard	Cereal	Legume	Horticultural	Fallow	Hops	Total
León (excl. El Bierzo)	7	13	10	1	7	3	41
León–El Bierzo	13	–	–	–	–	–	13
Zamora	1	2	–	1	1	–	5
Valladolid	2	–	–	–	2	–	4
Burgos	–	–	–	2	–	–	2
Asturias	–	–	–	–	1	–	1
Total	23	15	10	4	11	3	66

### 2.2 Soil parameters

Soil physicochemical properties were measured by the Instrumental Techniques Laboratory of the University of León (LTI, Universidad de León, Spain) external service according to official guidelines (Ministerio de Agricultura, 1976), assessing the soil pH, the OM content with the Walkley–Black method, total nitrogen (TKN) using the modified Kjeldahl method (International Organization for Standardization, 1995), and the C/N ratio, which was calculated as oxidizable carbon (Walkley–Black) divided by the TKN. Soil texture was assessed using the Bouyoucos hydrometer method and classified according to the United States Department of Agriculture (USDA) soil texture triangle (**Supplementary Table S1**).

### 2.3 Fungal isolation from soil samples

For fungal isolation, the method described in Mayo-Prieto et al. (2020) was followed. In brief, 100 µl aliquot of diluted soil suspensions was plated onto Rose Bengal chloramphenicol (RBC) agar medium (Conda Laboratory, Torrejón de Ardoz, Madrid, Spain). Two strains were directly cultured from plant tissue onto RBC. After 7 days of incubation at 25°C under 12-h light/darkness photoperiod, the originated fungal colonies, putatively identified as *Fusarium* species according to their macroscopic characteristics (Leslie and Summerell, 2006), were transferred into new potato dextrose agar (PDA) plates. A maximum of three selected distinct *Fusarium* isolates for each soil sample were purified and cultured on Spezieller Nährstoffarmer agar (SNA) medium (KH_2_PO_4_, 1 g/L; KNO_3_, 1 g/L; MgSO_4_·7H_2_O, 0.5 g/L; KCl, 0.5 g/L; sucrose, 0.2 g/L; glucose, 0.02 g/L; and agar, 20 g/L), sealed with Parafilm (Amcor, Neenah, WI, USA), and incubated for 12 days at room temperature (20–25°C) under natural light. Once grown, the SNA plates were observed directly under a microscope to determine *Fusarium* microscopic characteristics (Leslie and Summerell, 2006) and the monosporic cultures elaborated. Pure strains were stored at −80°C in 30% glycerol.

### 2.4 Molecular identification of the strains and phylogeny analysis

Preliminary identification was carried out at the Instrumental Techniques Laboratory (LTI) external service by sequencing the internal transcribed spacer (ITS) region and aligning to available data from the National Center for Biotechnology Information (NCBI) database using BLAST software (Camacho et al., 2009) to confirm that the selected strains belong to the genus *Fusarium*. For correct identification to the species level, the translation elongation factor 1α (*tef1*) gene, along with previously sequenced ITS regions, was selected among the most informative molecular markers.

For each *Fusarium* strain, the *tef1* gene was amplified and sequenced using the primer pair EF-1/EF-2 (O’Donnell et al., 1998). In brief, each strain was grown over cellophane disks on PDA Petri dishes; incubated for 3 days at 25°C; and the mycelium scraped, frozen, and lyophilized. DNA extraction was carried out using the “Wizard Magnetic DNA Purification System for food” kit (Promega Corporation, Madison, WI, USA) on 10–15 mg of powdered lyophilized mycelium. The DNA quantity and quality were assessed using Thermo-Scientific NanoDrop (LabX, Midland, ON, Canada) and agarose 0.8% gel electrophoresis in comparison with a standard DNA (1-kb DNA ladder; Thermo Fisher Scientific, Vilnus, Lithuania). Subsequent PCR reactions (total volume of 15 μl) were performed in the Mastercycler EP Gradient thermocycler (Eppendorf, Hamburg, Germany) following the protocol reported by Alijani Mamaghani et al. (2024) for *tef1*, and products stained with GelRed^®^ (GelRed^®^Nucleic Acid Gel Stain, 10,000×; Biotium Inc., Fremont, CA, USA) were checked after electrophoretic separation on 1.5% agarose gel in comparison with the 100-bp DNA ladder (Invitrogen, Thermo Fisher Scientific, Waltham, MA, USA). Each amplicon was purified with enzymatic mixture of exonuclease I/FastAP thermosensitive alkaline phosphatase (Thermo Fisher Scientific, Vilnius, Lithuania), and labeling was performed with the Big Dye Terminator v3.1 Cycle Sequencing Ready Reaction Kit (Applied Biosystems, Foster City, CA, USA). Labeled amplicons were purified by filtration through Sephadex G-50 (5%; Sigma-Aldrich, Saint Louis, MO, USA) and sequenced in “ABI PRISM 3730 Genetic Analyzer” (Applied Biosystems, Foster City, CA, USA).

The obtained FASTA sequences were analyzed and assembled using BioNumerics v.5.1 software (Applied Maths, Kortrijk, Belgium) and compared with the sequences available in the NCBI database through the BLASTN program.

In addition, the sequences obtained in this study along with 19 reference sequences of *Fusarium* were aligned using the MUSCLE algorithm (Edgar, 2004). Phylogenetic analysis was carried out using the maximum likelihood method with MEGA software version 7 (Kumar et al., 2016). All positions containing gaps and missing data were eliminated, considering a total of 448 positions in the final dataset. Bootstrap analysis (Felsenstein, 1985) was conducted to determine the confidence of internal nodes using a heuristic search with 1,000 replicates.

### 2.5 *In vitro* mycotoxin analysis

Parboiled rice (15.5 g) was hydrated with 7 ml of sterile water, autoclaved twice in 50-ml Falcon™ tubes (121°C for 20 min), and inoculated with three mycelial plugs (*Ø* = 4 mm) from the actively growing margin of colonies cultured on PDA plates. Three control strains (*F. oxysporum* f. sp. *lycopersici* CECT 2866, *Fusarium sporotrichoides* CECT 20166, and *Fusarium graminearum* CECT 2150) and uninoculated rice controls were included. The samples were incubated horizontally in a growth chamber at 25°C in darkness under 60% relative humidity for 3 weeks, shaken daily. After incubation, the rice cultures were dried at 45°C for 24 h and ground into a fine powder.

For mycotoxin extraction, 5 g of the powdered sample were weighed into 50-ml Falcon™ tubes and extracted with 20 ml acetonitrile/water/acetic acid (79:20:1, *v*/*v*/*v*) for 90 min on a rotary shaker at 170 rpm. The extracts were diluted with Milli-Q water (1:10, *v*/*v*) and directly injected into the LC-HRMS instrument (Streit et al., 2013).

LC-HRMS analysis was performed using an Orbitrap Exploris™ 240 instrument equipped with a heated electrospray ionization source (HESI II) coupled to a Vanquish UHPLC system (Thermo Fisher Scientific, San Jose, CA, USA).

Chromatographic separation was performed at 40°C on a Gemini^®^ C18-column (150 mm × 2 mm i.d., 5 μm particle size) equipped with a C18 (4 mm × 3 mm i.d.) security guard cartridge (Phenomenex, Torrance, CA, USA). The mobile phase consisted of water (eluent A) and methanol (eluent B), both containing 0.5% acetic acid and 1 mM ammonium acetate. The flow rate was 0.35 ml/min, and the injection volume was 10 µl. The gradient program was as follows: 10% B for 3 min, increased linearly to 50% B from 3 to 7 min and 90% B from 7 to 20 min, then to 100% B from 20 to 21 min, followed by a 6-min hold at 100% B. The system was then returned to the initial conditions (10% B) from 27 to 28 min and equilibrated for 7 min at 90% A.

The HESI II source was operated in both positive and negative ionization modes under the following conditions: sheath gas, 30 (arbitrary units); auxiliary gas, 5; sweep gas, 1; spray voltage, 3.5 kV (positive mode) and 2.5 kV (negative mode); RF lens, 70% (arbitrary units); ion transfer tube, 325°C; and vaporizer temperature, 300°C.

Full-scan high-resolution mass spectra were acquired in both ionization modes within a single run at a resolution of 120,000 over an *m*/*z* range of 100–850.

Mycotoxins were quantified by external calibration using a 1/*X* weighted regression model based on multi-component commercial standard solutions. Analytical standards included the following toxins: nivalenol (NIV), DON, 15-acetyl-deoxynivalenol (15-ADON), 3-acetyl-deoxynivalenol (3-ADON), neosolaniol (NEO), HT-2, T-2, fumonisins B_1_, B_2_, and B_3_ (provided as a mixed standard solution), and ZEA (Biopure™, Romer Labs, Getzersdorf, Austria); ENNs (A, A_1_, B, and B_1_), BEA, and diacetoxyscirpenol (DAS) (Libios, Fianovis, Vindy sur Turdine, France); and fusaric acid (≥99% purity; Supelco, Merck KGaA, Darmstadt, Germany). Fusarin C was determined semi-quantitatively due to the lack of availability of an analytical standard, but was included because of its known occurrence and toxicological relevance in *Fusarium* spp.

Peak areas were normalized to the sample exhibiting the highest signal, which was assigned a value of 1. The remaining samples were expressed as relative values.

The limits of detection (LOD) and quantification (LOQ) of each mycotoxin were calculated based on signal-to-noise ratios of 3 and 10, respectively, with noise measured in proximity to each analyte peak (Gialluisi et al., 2025). The LOQ values (in micrograms per kilogram) were 62.5 for NIV; 78.1 for DON; 18.8 for ADON; 12.5 for HT-2, T-2, and fusaric acid; and 6.3 for NEO, DAS, FB_1_, FB_2_, ZEA, ENN A, ENN A_1_, ENN B, ENN B_1_, and BEA.

The final concentrations were calculated by interpolating the peak areas on the calibration curves prepared in matrix (matrix-matched calibration) and applying the appropriate dilution factors, with the results expressed as micrograms per kilogram of sample.

### 2.6 Statistical analysis and graphics

R software v.4.4.2 (R Core Team, 2024) was used for all statistical analyses (seed = 123 for reproducibility), while graph generation used the packages ggplot2 (Wickham, 2016) and corrplot (Wei and Simko, 2024). Benjamin–Hochberg (BH) correction was applied to all multiple comparisons to compute the adjusted *p*-values (*p*_-adj_).

#### 2.6.1 Data preparation, transformation, and variable selection

Different subsets of the original dataset (*N* = 122) (**Supplementary Table S1**) were used according to the analysis performed.

- Isolated/identified species (*N* = 119): control strains excluded.
- Mycotoxin characterization (*N* = 115): strains with valid mycotoxin measurement, re-including the control strains and removing seven unmeasured samples.
- A binary transformation (presence/absence) of these data was applied for detection analysis.
- Univariate and multivariate analyses of the toxigenic profiles (*N* = 110): strains with complete metadata, removing the control strains and two plant-derived samples lacking soil parameters.
- For the multivariate analysis, the toxin matrix was Hellinger-transformed, and the Euclidean (Hellinger) distances were calculated after removing the samples that did not produce any of the quantified toxins, resulting in *N* = 107.

Accordingly, the following variables were also selected:

- Dependent variables (toxin) and constant zero columns (NIV: undetected; ADON and T-2: produced only by the controls) were removed from the univariate and multivariate analyses. Fusarin C, which was measured semi-quantitatively, was excluded from the multivariate distance-based methods.
- Independent variables (metadata) with more than 10% missing data (e.g., crop rotation, soil texture, and coordinates, among others) and variables with singleton levels, confounding, or high collinearity (Pearson’s *r* > 0.7) were not taken into account. Therefore, the retained variables were species complex (SC), soil OM, soil pH, soil C/N ratio, and associated crop.

#### 2.6.2 Mycotoxin production characterization

The species and toxin production data were summarized using dplyr (Wickham et al., 2023). A heatmap was generated using the binary transformed toxin matrix, showing the proportions (in percent) of strains producing each mycotoxin relative to the total number of strains of the same species. Spearman’s rank correlations were used to quantify the relationships between toxin pairs and significant associations (*p_-_*_adj_ < 0.05) displayed with corrplot.

#### 2.6.3 Toxigenic profile structure and species complex

Euclidean distances from the Hellinger-transformed mycotoxin production matrix (vegan) (Oksanen et al., 2025) were used for all distance-based multivariate analysis.

- Principal coordinates analysis (PCoA) was used to ordinate the strains according to their similarity regarding the whole toxigenic profile. Mycotoxin vectors were fitted onto the PCoA space using envfit (vegan, 999 permutations), and only the significant ones (*p*_-adj_ < 0.05) are displayed.
- For clustering, the number of clusters was explored using the silhouette method (factoextra) (Kassambara and Mundt, 2020), and cluster (*k*-means) stability was assessed by bootstrap resampling (fcp, Jaccard index) (Hennig, 2026). Cluster assignation was examined against species, SC, and metadata, applying Fisher’s test with Monte Carlo simulation for discrete variables and the Wilcoxon rank-sum test for continuous variables.
- Permutational multivariate analysis of variance (PERMANOVA) was computed with adonis2 (vegan, 999 permutations) to examine the differences in the whole mycotoxin profile among SCs, and homogeneity of dispersion was assessed with betadisper.
- Kruskal–Wallis tests (Ogle et al., 2025) were used to examine differences in the individual toxins across SCs, with effect sizes estimated as *η*^2^ (eta2, rstatix) (Kassambara, 2025) followed by Dunn’s *post-hoc* test (FSA) (Ogle et al., 2025).

#### 2.6.4 Association with soil parameters

Transformation-based redundancy analysis (tb-RDA) conditioning on SC was performed on the Hellinger-transformed data to determine whether the selected independent variables structured the toxigenic profile beyond taxonomic identity. Significance was assessed by permutation (999 permutations, marginal terms), the explained fraction of variation by adjusted *R*^2^, and the collinearity among constraints by variance inflation factors (VIFs). Differences in the soil parameters across SCs were further examined using Kruskal–Wallis tests.

## 3 Results

### 3.1 Occurrence of *Fusarium* species in soil samples and mycotoxin detection

All soil samples were contaminated by *Fusarium* spp., except for three soil samples collected in three different vineyards: two from Valladolid and one from El Bierzo, León. A total of 160 *Fusarium* strains were isolated from 63 of the 66 total soil samples collected from different crop fields located in the northwestern part of Spain and two directly from plant tissue. In order to obtain a clear picture of the *Fusarium* spp. present in the monitored fields, a maximum of three strains showing different morphological traits were collected for each soil sample, and preliminary identification was confirmed by sequencing of the ITS region. From this initial set, we retrieved the same species’ strains from the same soil samples, resulting in 119 strains. These were subjected to identification to the species level using *tef1* sequencing (O’Donnell et al., 2015). Only one sample (F-S086) could not be properly identified to the species level, which was then reported as *Fusarium* sp. from the *Fusarium incarnatum–equiseti* species complex (FIESC) according to its ITS sequence, which allows resolution to the SC level (O’Donnell et al., 2022) in **Table 2**.

**Table 2:** Summary of the *Fusarium* species isolated in this work.

Species	Species complex	Percentage	Number
*F. algeriense*	FBURSC	1.68	2
*F. proliferatum*	FFSC	1.68	2
*F. subglutinans*	FFSC	0.84	1
*F. temperatum*	FFSC	1.68	2
*F. clavum*	FIESC	2.52	3
*F. equiseti*	FIESC	6.72	8
*F. flagelliforme*	FIESC	0.84	1
*F. scirpi*	FIESC	0.84	1
*Fusarium* sp.^a^	FIESC	0.84	1
*F. nirenbergiae*	FOSC	10.92	13
*F. oxysporum*	FOSC	35.29	42
*F. redolens*	FRSC	4.20	5
*F. brachygibbosum*	FSAMSC	5.88	7
*F. solani*	FSSC	23.53	28
*F. vanettenii*	FSSC	1.68	2
*F. torulosum*	FTSC	0.84	1

Shown are the *Fusarium* species, the species complex (SC) to which they belong, the percentage of each species in the total isolated strains (*N* = 119), and the number of strains.

FBURSC, *Fusarium burgessii* SC; FFSC, *Fusarium fujikuroi* SC; FIESC, *Fusarium incarnatum–equiseti* SC; FOSC, *Fusarium oxysporum* SC; FRSC, *Fusarium redolens* SC; FSAMSC, *Fusarium sambucinum* SC; FSSC, *Fusarium solani* SC; FTSC, *Fusarium tricinctum* SC.

^a^ F-S086 strain identified only to the SC level.

### 3.2 Molecular identification of *Fusarium* species and phylogenetic analysis

Phylogenetic analysis of the *tef1* nucleotide sequences involved a set of 118 *Fusarium* strains isolated in this study (excluding strain F-S086 identified only to the SC level and the control strains) and 19 *Fusarium* reference sequences. The analysis allowed grouping all strains into eight well-separated clades, supported by high bootstrap values, corresponding to *Fusarium oxysporum* SC (FOSC), *Fusarium fujikuroi* SC (FFSC), *Fusarium redolens* SC (FRSC), *Fusarium burgessii* SC (FBURSC), *Fusarium tricinctum* SC (FTSC), *Fusarium solani* SC (FSSC), *Fusarium sambucinum* SC (FSAMSC), and *F. incarnatum–equiseti* SC (FIESC). A summary of the identified species is reported in **Table 2**, along with the *Fusarium* sp. strain F-S086 corresponding to FIESC. The phylogenetic tree is displayed in **Figure 1**.

**Figure 1:**
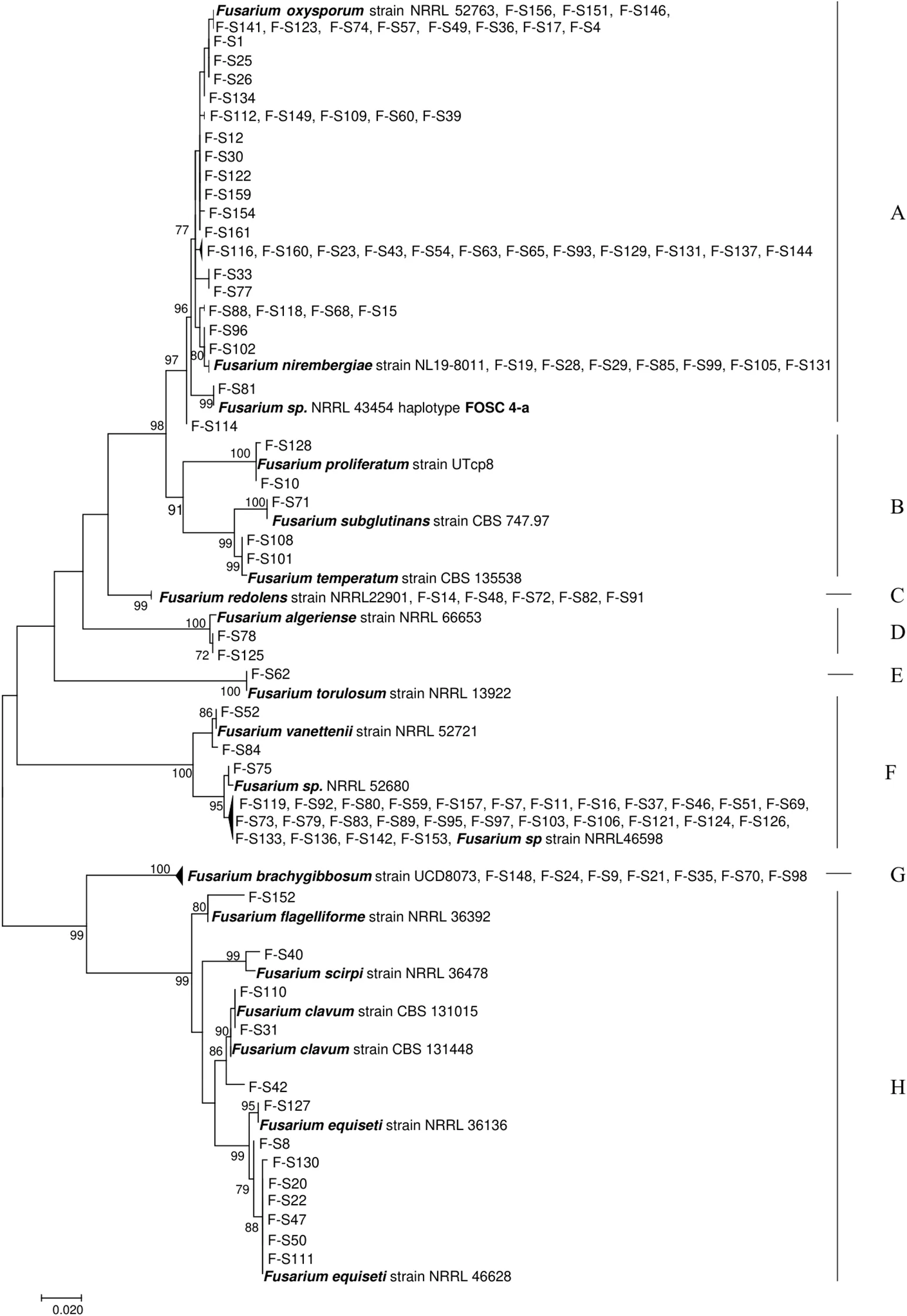
Phylogenetic tree of the isolated strains. According to the *tef1* sequence of the 118 strains successfully identified in this study plus 19 reference sequences, a phylogenetic tree based on the maximum likelihood method (bootstrap 1,000) was constructed. Bootstrap support values (in percent) are shown at the *nodes*. Strain codes were arbitrarily assigned by the order of isolation and correspond to the “sample code” in **Supplementary Table S1**. *Letters on the right side* indicate distinct clades constituting the species complexes (SC) defined. **(A)** *Fusarium oxysporum* SC. **(B)** *Fusarium fujikuroi* SC. **(C)** *Fusarium redolens* SC. **(D)** *Fusarium burgessii* SC. **(E)** *Fusarium tricinctum* SC. **(F)** *Fusarium solani* SC. **(G)** *Fusarium sambucinum* SC. **(H)** *Fusarium incarnatum–equiseti* SC.

Majority of the strains (46.6%) clustered together and with the reference strains *F. oxysporum* NRRL 52763, *Fusarium nirenbergiae* NL19-8011, and *Fusarium* sp. NRRL43454 haplotype FOSC 4a (clade A, **Figure 1**). In detail, 40 strains showed high homology to *F. oxysporum* NRRL52763, 13 strains grouped with the *F. nirenbergiae* reference strain, and strain F-S081 showed 100% homology to the haplotype FOSC 4a. The homology of F-S114 to *F. oxysporum* was confirmed via BLASTN, making a total of 55 FOSC members (**Table 2**).

Only five strains grouped in clade B, namely, FFSC, among which F-S010 and F-S128 were identified as *Fusarium proliferatum*, F-S071 was identified as *Fusarium subglutinans*, and two strains, F-S101 and F-S108, were identified as *Fusarium temperatum*. Five strains collected from different soil samples—F-S014, F-S048, F-S072, F-S082, and F-S091—showed 100% homology among them and to *F. redolens* NRRL46685, which was used as a reference strain (clade C). Only two strains, F-S078 and F-S125, were identified as *Fusarium algeriense* clustering with the reference strain NRRL66653 (clade D). Clade E consisted of the strain F-S062 and *Fusarium torulosum* NRRL13922, member of FTSC. Out of 118 strains, 30 were identified as members of FSSC, which clustered together (clade F) with *Fusarium vanettenii* NRRL52721, *F. solani* NRRL52680, and *F. solani* NRRL46598 reference sequences. In particular, except for two strains (F-S052 and F-S084) showing high homology to *F. vanettenii* species and a single strain (F-S075) that grouped with *F. solani* NRRL52680, all strains, which showed low variability among them, grouped with the *F. solani* NRRL46598 reference strain (clade F). Seven strains clustered with the *Fusarium brachygibbosum* UCD8073 reference strain (clade G). A high variability was observed among the members of FIESC (clade H). Six out of 13 FIESC strains showed 100% homology to *F. equiseti* NRRL46628, a single strain (F-S127) showed 100% homology to *F. equiseti* NRRL36136, and three strains (F-S031, F-S042, and F-S110) clustered with the *Fusarium clavum* CBS 131015 and CBS 131448 reference strains. Two strains (F-S152 and F-S040) included in this clade were molecularly identified as *Fusarium flagelliforme* and *Fusarium scirpi*, respectively, and a single strain (F-S040) as *F. scirpi*.

### 3.3 Mycotoxin production

The results of the mycotoxin measurements via LC-HRMS analysis are summarized in **Table 3**, which reports descriptive statistics for each analyte and is described in the next subsection. Three control strains were added (*F. oxysporum* CECT 2866, *F. sporotrichoides* CECT 20166, and *F. graminearum* CECT 2150) to include known producers of trichothecenes and a well-characterized *F. oxysporum* strain, which was our most numerous species, while strains F-S001, F-S031, F-S042, F-S062, F-S089, F-S119, and F-S152 could not be properly measured for mycotoxin production and were subsequently removed from this analysis.

**Table 3:** Descriptive statistics of the mycotoxin production.

Toxin	*N* sample	*n* positive	% positive	Mean (mg/kg)	Median (mg/kg)	SD	Min. (mg/kg)	Max. (mg/kg)
NIV	115	0	0	–	–	–	–	–
DON	115	3	2.61	16.79	2.74	25.28	1.66	45.98
ADON	115	1	0.87	5.04	5.04	–	5.04	5.04
NEO	115	11	9.57	55.90	11.32	87.41	0.10	240.30
DAS	115	11	9.57	1.51	0.31	2.50	0.03	7.89
HT-2	115	4	3.48	5.69	0.14	11.10	0.14	22.33
T-2	115	1	0.87	89.31	89.31	–	89.31	89.31
FB_1_	115	4	3.48	227.67	1.33	453.47	0.15	907.86
FB_2_	115	3	2.61	62.14	0.43	107.07	0.23	185.78
ZEA	115	10	8.70	58.73	0.22	181.15	0.03	574.25
ENN B	115	39	33.91	68.90	4.39	180.09	0.03	976.00
ENN B_1_	115	39	33.91	101.21	8.04	228.31	0.03	986.41
ENN A_1_	115	51	44.35	189.23	0.53	479.00	0.03	2,470.57
ENN A	115	52	45.22	1027.81	2.72	2,654.31	0.14	13,528.21
BEA	115	105	91.30	474.63	61.12	670.87	0.03	2,379.71
Fusaric acid	115	32	27.83	0.68	0.68	0.32	0.06	1.34
Fusarin C^a^	115	24	20.87	0.20	0.10	0.24	0.01	1.00

For each measured mycotoxin, the total number of samples (*N* samples), the number of positive samples (*n* positives), and the percentage of the total they represent (% positives) are reported. The mean, median (in milligrams per kilogram), standard deviation (SD), and the lowest (Min.) and highest (Max.) concentrations (in milligrams per kilogram) are reported for positive samples.

NIV, nivalenol; DON, deoxynivalenol; ADON, acetyl-deoxynivalenol; NEO, neosolaniol; DAS, diacetoxyscirpenol; HT-2, HT-2 toxin; T-2, T-2 toxin; FB_1_, fumonisin B_1_; FB_2_, fumonisin B_2_; ZEA, zearalenone; ENN, enniatin (B, B_1_, A_1_, and A); BEA, beauvericin.

^a^ Calculations for fusarin C were done with the semi-quantitative (relative 0–1 scale) data.

#### 3.3.1 Description of mycotoxin data

BEA was the most frequently detected mycotoxin, occurring in 91% of the samples (105/115), followed by ENNs, with detection frequencies ranging from 34% to 45%. Fusaric acid and fusarin C were detected in 28% and 21% of the samples, respectively, while NIV was not detected in any sample. In terms of concentration, ENNs and BEA showed the highest production levels. The mean total ENN concentrations ranged from 6.9 mg/kg (ENN B) to 102.8 mg/kg (ENN A), while BEA reached a mean concentration of 47.5 mg/kg. Regardless of its prevalence, fusaric acid was produced at low concentrations, reaching a mean value of 0.7 mg/kg, while fusarin C was not measured in absolute values. In contrast, other mycotoxins were detected at lower frequencies (less than 10%): the trichothecenes ADON and T-2 were only produced by the control strains at concentrations of 0.5 and 8.9 mg/kg, respectively, followed by other more frequently detected trichothecenes, but always at lower concentrations than that of T-2. Fumonisins were seldom produced, but at relatively high mean concentrations (22.8 and 6.2 mg/kg for FB_1_ and FB_2_, respectively) (**Table 3**).

High standard deviations reflected the large variability in the mycotoxin concentrations and the absence of production in a substantial proportion of the samples, indicating a zero-inflated non-normal distribution.

#### 3.3.2 Mycotoxin detection

By transforming the production data into a binary dataset of presence/absence, a heatmap was elaborated to visualize the percentage of strains of each species that produced each mycotoxin, along with the sample number and SC to which they belong. This does not take the concentration values into account, and high detection rates do not necessarily imply co-production with *n* > 2 (**Figure 2**). BEA was detected with high frequency in almost every species, with the exception of *F. subglutinans* and *Fusarium* sp. FIESC members. The four ENNs were detected in all strains of *F. algeriense*, *F. redolens*, and the control strain *F. oxysporum* CECT 2866, while *F. nirenbergiae*, *F. oxysporum*, *F. brachygibbosum*, and *F. solani* displayed lower detection rates. Different combinations of ENNs were detected in *F. equiseti* and *F. scirpi* (all but ENN B), *F. proliferatum* (ENN A and ENN A_1_), and the control strains from FSAMSC (only ENN A_1_). Fusaric acid was produced at moderate to low rates (high for species with *n* < 2) for all SCs but FFSC, and fusarin C was mainly detected in FSSC at moderate rates and in both *F. algeriense* strains. Apart from the control strain *F. graminearum*, ZEA was detected in approximately 75% of the *F. equiseti* strains (and the *Fusarium* sp. strain), *F. proliferatum*, and *F. algeriense*. Both FBs were frequently produced by *F. proliferatum* (*n* = 2), but no other FFSC members. The trichothecenes, apart from that in the FSAMSC control strains, were only detected in *F. brachygibbosum* (DAS and NEO produced by all strains), FIESC members, and *F. algeriense* at lower rates.

**Figure 2:**
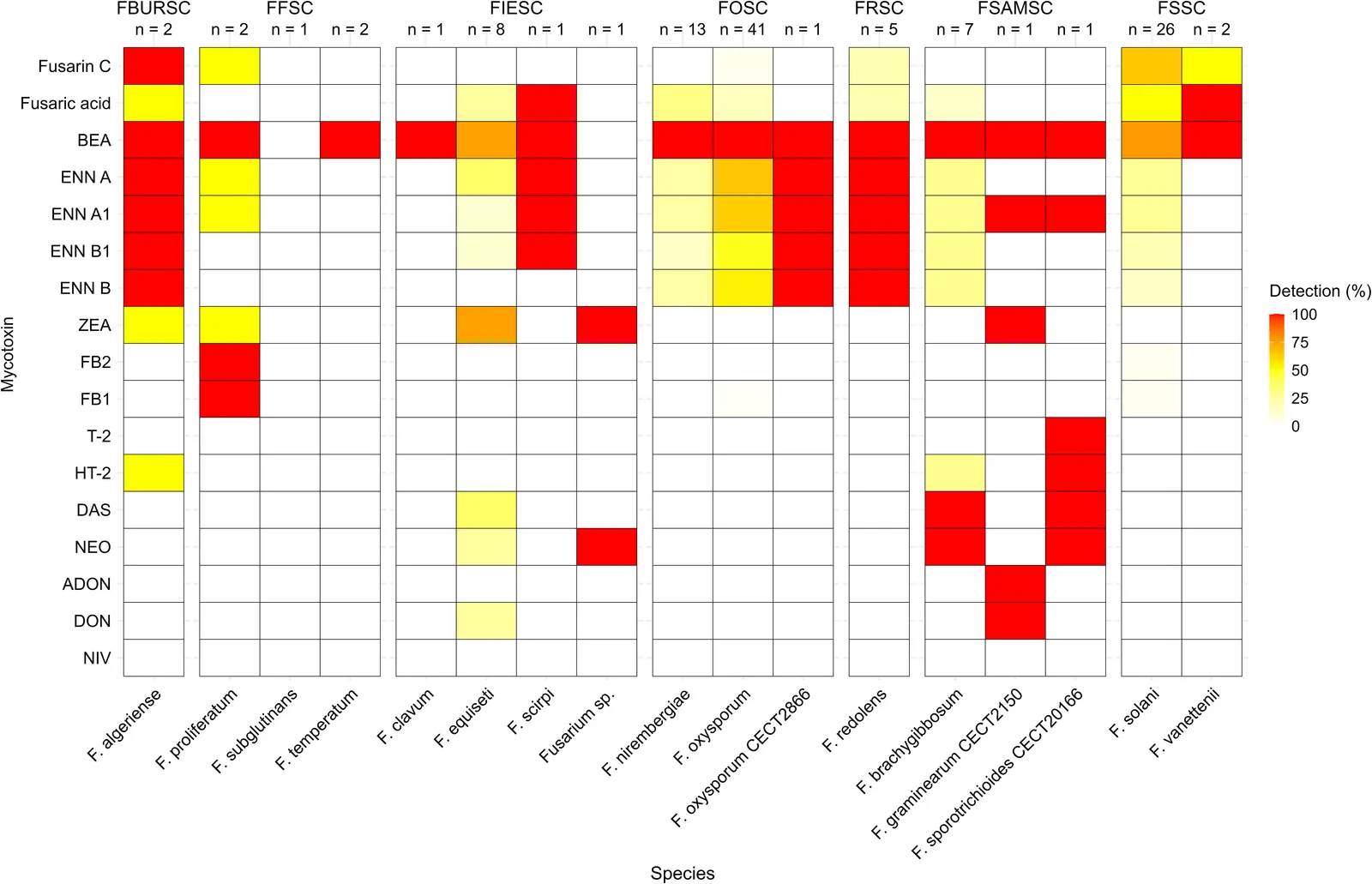
Heatmap of the mycotoxin detection percentage per species. Toxins are displayed on the *Y*-axis and the species name on the *X*-axis (control strains with their corresponding code). NIV, nivalenol; DON, deoxynivalenol; ADON, acetyl-deoxynivalenol; NEO, neosolaniol; DAS, diacetoxyscirpenol; HT-2, HT-2 toxin; T-2, T-2 toxin; FB_1_, fumonisin B_1_; FB_2_, fumonisin B_2_; ZEA, zearalenone; ENN, enniatin (B, B_1_, A_1_, and A); BEA, beauvericin. Panels are organized by species complex (SC): *FBURSC*, *Fusarium burgessii* SC; *FFSC*, *Fusarium fujikuroi* SC; *FIESC*, *Fusarium incarnatum–equiseti* SC; *FOSC*, *Fusarium oxysporum* SC; *FRSC*, *Fusarium redolens* SC; *FSAMSC*, *Fusarium sambucinum* SC; *FSSC*, *Fusarium solani* SC. The total count of each species is displayed *on top of each panel* as “*n*”. The toxin detection percentage (frequency of detection/total number of strains of the species) using a binary transformation of the data (*n* = 115) for presence/absence is indicated by the *color gradient*. *Orange* to *red cells* represent a high percentage (50%–100%) of isolates from a certain species producing the mycotoxin, while *yellow* to *white cells* indicate low percentage (50%) to absence (0%) of detection of strains producing the toxin.

#### 3.3.3 Mycotoxin correlation

To reveal mycotoxin co-production, we calculated Spearman’s non-parametric correlations between the toxin’s absolute production data and retained only the significant ones (*p*_-adj_ < 0.05) (**Figure 3**). In terms of the concentration patterns, all ENNs correlated positively with each other (*ρ* between 0.86 and 0.95) and with BEA (*ρ* between 0.39 and 0.43). BEA production, apart from the positive correlation with ENNs, had a negative relationship with ZEA (*ρ* = −0.31), meaning that the strains that produced BEA tended to not produce ZEA. ZEA correlated positively with DON and ADON (*ρ* = 0.34 and 0.33, respectively). The type A trichothecenes (NEO, DAS, HT-2, and T-2) all correlated positively among themselves, with coefficients ranging from 0.30 (DAS *vs*. T-2) to 0.91 (NEO *vs*. DAS). Similarly, DON and ADON were also positively associated (*ρ* = 0.58), and DON correlated with DAS (*ρ* = 0.31). The fumonisin pair FB_1_ and FB_2_ were strongly correlated (*ρ* = 0.87). Fusaric acid and fusarin C showed a positive association (*ρ* = 0.35), and both were negatively correlated with BEA production (*ρ* = −0.27 and −0.33).

**Figure 3:**
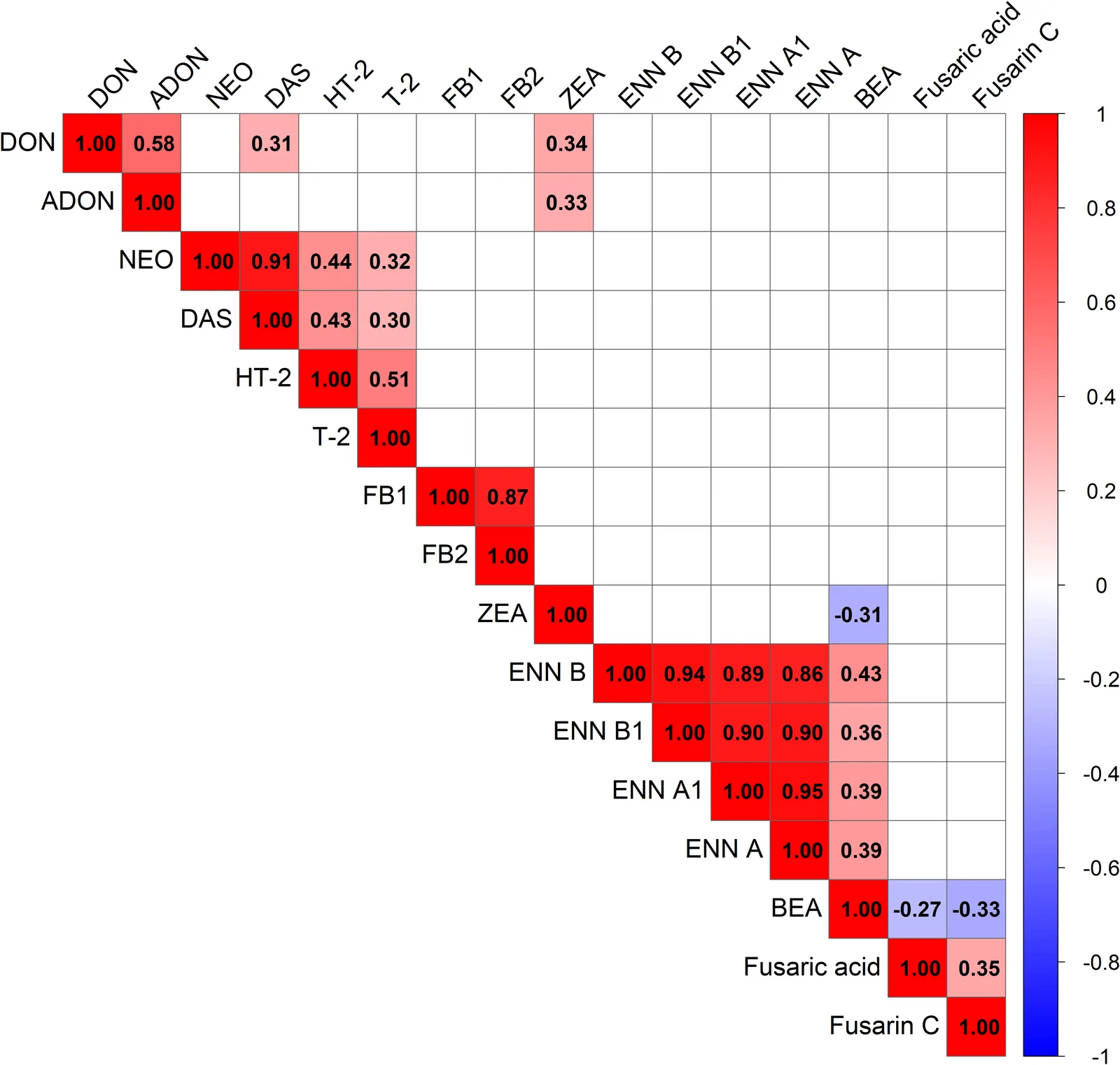
Significant Spearman’s correlation coefficients among mycotoxins. Calculated from absolute production data, removing nivalenol (NIV; no production). Only statistically significant correlations are shown (*p_-_*_adj_ < 0.05). *Red* indicates high Spearman’s coefficients (*ρ*), indicating positive correlations in the toxin production concentration, while *blue* indicates negative coefficients. Abbreviation: DON, deoxynivalenol; ADON, acetyl-deoxynivalenol; NEO, neosolaniol; DAS, diacetoxyscirpenol; HT-2, HT-2 toxin; T-2, T-2 toxin; FB1, fumonisin B1; FB2, fumonisin B2; ZEA, zearalenone; ENN, enniatin (B, B1, A1, and A); BEA, beauvericin.

### 3.4 Mycotoxin production profile is driven by intrinsic factors

In order to reduce the dimensionality of the data and explore patterns that might be occurring, PCoA was carried out. Three strains (F-S007, F-S069, and F-S071) did not produce any of the quantified toxins and were therefore removed from the ordination (Legendre and Gallagher, 2001).

The PCoA (**Figure 4**) of the Hellinger-transformed (Euclidean distance) toxigenic profiles captured 47.9% and 18.8% of the total variation in PCoA1 and PCoA2, respectively. Significant mycotoxin vectors fitted onto the ordination (envfit: *p*_-adj_ < 0.05), indicating that BEA loaded mainly on PCoA1 (to the upper left); ENNs contributed to both axes (to the upper right diagonally), with ENN A being the largest vector; and fusaric acid (downward right) and NEO and ZEA (with smaller vectors pointing downward) contributed almost exclusively to PcoA2. Individual strains (colored by SC) were distributed in a continuous cloud around the origin. FOSC (brown) and FRSC (yellow) were spread mainly in the upper part, following the BEA–ENNs axis, while FSAMSC (light blue) appeared between the arrows from BEA to NEO/ZEA. FSSC (green) and FIESC (dark blue) were more dispersed along the downward right part of the ordination, following the NEO/ZEA–fusaric acid–ENN pull. Less represented SCs appeared interspersed in the central part of the ordination, suggesting less differentiated profiles.

**Figure 4:**
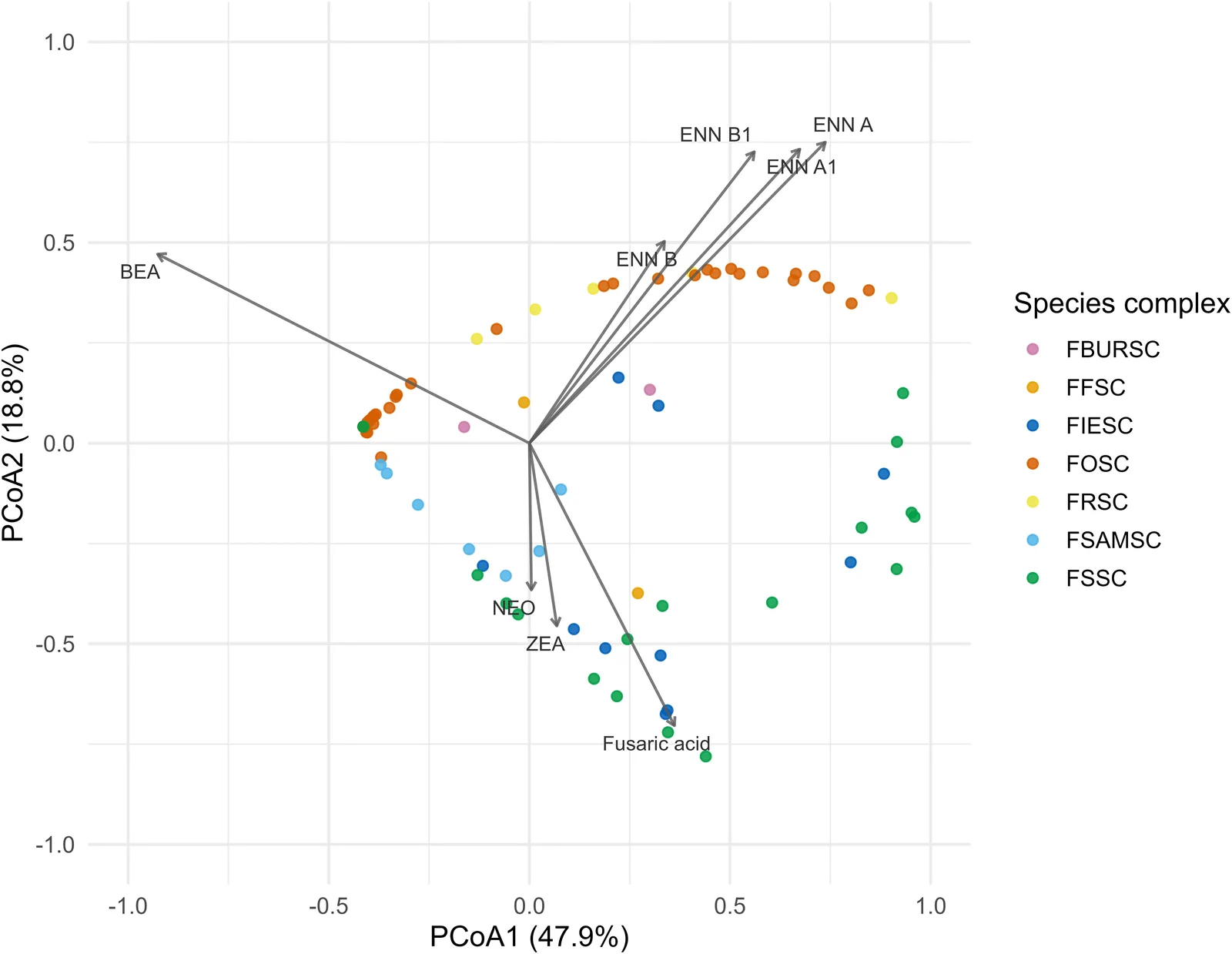
Representation of the principal coordinates analysis (PCoA) of Hellinger-transformed (Euclidean distance) toxin data (*n* = 107). PCoA1 and PCoA2 represent the main axis of variation, and significant mycotoxin vectors (envfit: *p*_-adj_ < 0.05) were fitted on the ordination. BEA, beauvericin; ENN, enniatin; ZEA, zearalenone; NEO, neosolaniol. Dots indicate individual strains, colored by the species complex (SC) to which they belong. *FBURSC*, *Fusarium burgessii* SC (*pink*); *FFSC*, *Fusarium fujikuroi* SC (*orange*); *FIESC*, *Fusarium incarnatum–equiseti* SC (*dark blue*); *FOSC*, *Fusarium oxysporum* SC (*brown*); *FRSC*, *Fusarium redolens* SC (*yellow*); *FSAMSC*, *Fusarium sambucinum* SC (*light blue*); *FSSC*, *Fusarium solani* SC (*green*).

To assess whether toxigenic profiles with unclear grouping in the PCoA space constituted discrete groups, *k*-means clustering was carried out, evaluating two to eight clusters with bootstrap resampling. Although the silhouette method suggested six clusters, only the two-cluster partitioning was stable (Jaccard’s index = 0.89 and 0.93) (**Supplementary Table S2**, part A), indicating that the data did not support higher partitioning. These two clusters did not separate clusters according to total production, which did not differ between groups (Kruskal–Wallis: *p* = 0.69, not shown), but for the qualitative composition of the profile. The median strain of cluster 1 produced only one toxin, whereas that of cluster 2 showed a median production of five toxins (**Supplementary Table S2**, part B). The median of each toxin’s concentration per cluster showed that the first produced BEA at high concentrations (7 mg/kg), while the second produced all ENNs, fusaric acid, and BEA at lower concentrations (**Supplementary Table S2**, part C), meaning that at least half of the strains in cluster 2 produced these mycotoxins. Species identity and SC were statistically associated with cluster membership (Fisher’s exact test: *p*_-adj_ = 0.0075), while the other parameters (i.e., OM, pH, C/N, and associated crop) were not (Wilcoxon: *p*_-adj_ > 0.2) (**Supplementary Table S2**, part D). Regarding assignation to each cluster, species were rather divided into one or the other, except for the more abundant ones (*F. oxysporum*: 27 *vs*. 14; *F. solani*: 13 *vs*. 11) that were more evenly distributed. The same tendency was observed for SC (**Supplementary Table S2**, parts E and F).

As suggested by the SC partial grouping on the PCoA, PERMANOVA confirmed that SC was associated with the differences in the whole toxigenic profile (*p* = 0.001, *R*^2^ = 0.29, pseudo-*F* = 6.78) (**Table 4**). Multivariate dispersion also differed among SCs (betadisper: *p* = 0.002).

**Table 4:** Effect of species complex (SC) on the whole toxigenic profile.

Term	*df*	SumSq	*R* ^2^	pseudo-*F*	*p*	Significance
Model	6	13.5438803	0.28904991	6.77614177	0.001	***
Residual	100	33.3126648	0.71095009			
Total	106	46.8565451	1			

Permutational multivariate analysis of variance (PERMANOVA) based on Euclidean distances (Hellinger-transformed mycotoxin data, 999 permutations). *df* reports the degrees of freedom, *SumSq* the sum of squares, *R^2^* the proportion of variance explained, “pseudo-*F*” the test statistic, “*p*” the permutational *p*-value, and *Significance* summarizes the *p*-value significance (***p > 0.001). Multivariate dispersion among species complexes was heterogeneous (PERMDISP/betadisper: *p* = 0.002), indicating that the differences reflect both profile composition and dispersion.

To examine whether other variables influenced the mycotoxin profile, tb-RDA was performed, conditioning by SC, according to previous results showing the significance of this variable over toxin profiles and almost 30% of the variance explained by it (**Table 4**, *R*^2^). The tested predictors only explained 6% of the variance, and residual variance was 65% after conditioning by SC (30% variance) (**Supplementary Table S3**, part A). Neither the soil parameters (OM, pH, and the C/N ratio) nor the associated crop of the original soils explained a significant fraction of the variation in mycotoxin production (*p* > 0.05, *R*^2-adj^ = 0.006) (**Supplementary Table S3**, parts B and C).

Individually, all mycotoxins varied significantly across SCs (Kruskal–Wallis: *p*_-adj_ < 0.05) (**Supplementary Table S4**). Dunn’s *post-hoc* comparisons (excluding FBURSC due to the small sample size) revealed that, for the less prevalent mycotoxins (i.e., FBs, DON, HT-2, NEO, DAS, and ZEA), these differences reflected production restricted to single SCs rather than graded differences in concentration. The effect sizes were large (*η*^2^ > 0.14) for the majority of mycotoxins, indicating that SC explains a high proportion of the variation in individual mycotoxin production. Some toxins combined large effect sizes with small prevalence (NEO and DAS: *η*^2^ > 0.75, produced by 9.1% of the strains), while BEA and ENNs had high effect sizes and high prevalence.

Consistent with SC influencing the whole toxigenic profile and the individual differences in the mycotoxins shown above, the concentration ranges of the toxins were plotted in a box plot per SC (**Figure 5**, logarithmic scale) to summarize and visualize these differences.

**Figure 5:**
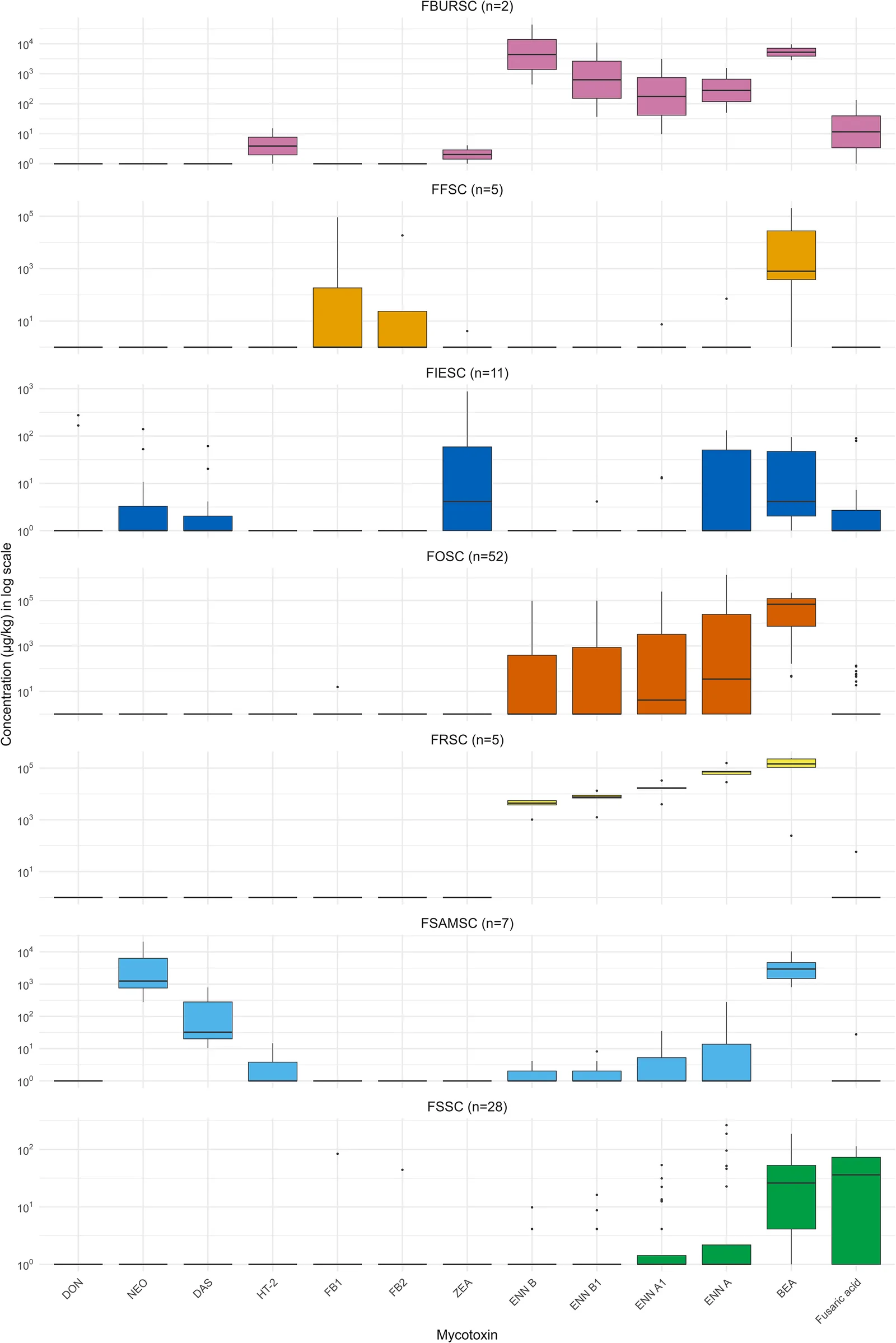
Box plot representing the mycotoxin profile per species complex (SC). Concentrations (in micrograms per kilogram) are displayed in logarithmic scale (log + 1) to compress distribution, and values are adjusted for each toxin’s production range (*N* = 110, non-transformed). Each *panel* represents one SC, and the number of samples is indicated by “*n*”. *FBURSC*, *Fusarium burgessii* SC; *FFSC*, *Fusarium fujikuroi* SC; *FIESC*, *Fusarium incarnatum–equiseti* SC; *FOSC*, *Fusarium oxysporum* SC; *FRSC*, *Fusarium redolens* SC; *FSAMSC*, *Fusarium sambucinum* SC; *FSSC*, *Fusarium solani* SC; DON, deoxynivalenol; ADON, acetyl-deoxynivalenol; NEO, neosolaniol; DAS, diacetoxyscirpenol; HT-2, HT-2 toxin; T-2, T-2 toxin; FB_1_, fumonisin B_1_; FB_2_, fumonisin B_2_; ZEA, zearalenone; ENN, enniatin (B, B_1_, A_1_, and A); BEA, beauvericin.

## 4 Discussion

### 4.1 Species composition of the collection

In the present article, we isolated and selected 119 *Fusarium* spp. strains from agricultural soils to determine their species identity and mycotoxin production profiles.

The dominant SCs in our collection were *F. oxysporum* SC, *F. solani* SC, and *F. incarnatum–equiseti* SC, consistent with their reported higher occurrence in soils (Saremi and Saremi, 2013; Edel-Hermann et al., 2015). In terms of individual species, 35% of the strains were *F. oxysporum*, followed by *F. solani* (24%) and *F. nirenbergiae* (11%), belonging to FOSC. The latter is of relatively recent description (Lombard et al., 2019) and has been observed to be associated with plant disease in a wide range of hosts and locations (Zhao et al., 2019; Aiello et al., 2021; López-Moral et al., 2024; Fallahi et al., 2025; Zhang et al., 2025). Nonetheless, to our knowledge, it has not yet been isolated directly from soil, and we can confirm the presence of this potential pathogen in Spanish agricultural soils, as suggested by the capacity of *Fusarium* spp. to persist in soil and plant debris (Nikitin et al., 2023). We also found other minoritarian *Fusarium* spp.: *F. algeriense* (two isolates, FBURSC) described by Laraba et al. (2017) as causing crown rot and head blight (Belabed et al., 2025) in Algerian wheat. To our knowledge, *F. algeriense* has not been reported in Europe. *F. clavum* (three isolates, FIESC) has been recently described by Xia et al. (2019) and linked to plant infection in Mediterranean areas (Gilardi et al., 2021; Narduzzi et al., 2026), while *F. flagelliforme* (one isolate, FIESC), also described by Xia et al. (2019) as associated with cereals in Europe, counts on limited reports, but might have a broader distribution (Xu et al., 2022). *F. temperatum* (two isolates, FFSC) has been reported in northwest Spain by Pintos Varela et al. (2013), consistent with our findings. *F. brachygibbosum* has been reported as a concerning potential pathogen in the EU (Bragard et al., 2021) and has only been described in Italy. Of the above, the latter is the only one present in the EPPO database (European and Mediterranean Plant Protection Organization, 2026).

### 4.2 Mycotoxin occurrence and coproduction

We expected mycotoxin production to be related to the species identity of the strains due to the differential biosynthetic clusters they possess based on the existing literature (Qu et al., 2024; Wang et al., 2024; Bugingo et al., 2025). Regarding toxin production by species (**Figure 2**), some surprising results were found for ENN production, which are not described for *F. algeriense*, *F. solani*, *F. nirenbergiae*, *F. temperatum*, *F. graminearum*, or *F. proliferatum*. In line with Munkvold et al. (2021), the FFUJ members *F. subglutinans* and *F. temperatum* did not produce FBs and the latter produced BEA, while *F. proliferatum* produced the expected FBs, ENNs, fusarin C, and BEA, along with ZEA (under LOQ, and thus this potential should be confirmed as it is not documented) and showing no production of fusaric acid. *F. equiseti* (FIESC) has been described to display a variety of gene clusters including trichothecenes, ZEA, ENNs, or fusarins (Villani et al., 2019), which aligns with our results, and *F. brachygibbosum* from FSAMSC producing trichothecenes (Bragard et al., 2021). Surprisingly, one *F. solani* strain (F-S011) (**Supplementary Table S1**) produced FBs, although FSSC is not considered as an FB producer (Munkvold et al., 2021; Qu et al., 2024). However, this specific strain may have functional FB biosynthetic genes, as suggested in some reports (Gamboa-Becerra et al., 2021). Interestingly, F-S010 was isolated from the same soil sample as the FB producer *F. proliferatum* (F-S010), and another *F. oxysporum* (F-S012) producing a small amount of FB_1_ was isolated from a nearby area belonging to the same farmer. This opens the door for further investigation on horizontal gene transfer and biosynthetic gene clusters in these strains.

In general, the observed production aligned with previous reports, with ENNs, BEA, and fusaric acid being detected more frequently than expected and FBs and trichothecenes less frequently detected than in the literature cited above. This may be due to “emerging” mycotoxins being less studied and not routinely tested for in food and feed contamination, although previous studies have shown the co-occurrence of ENNs (Stanciu et al., 2017; Xu et al., 2024), while FBs and trichothecenes may be more linked to plant infection of floral parts and roots and may not have been synthesized at high levels in our *in vitro* conditions (Desjardins et al., 2000; Perochon and Doohan, 2024). The majority of studies focused on the mycotoxin content in infected crops, while the soil isolation approach may produce patterns that differ from those commonly reported, as *Fusarium* spp. can survive as saprophytic fungi in the soil for several years (Todorović et al., 2023) and *in vitro* rice culture itself may also affect the mycotoxin production profile (Inbaia, 2023). Moreover, for some of the less studied species, data availability on mycotoxin production is limited.

Regarding the Spearman’s correlations among toxins (**Figure 3**), the positive correlation among ENNs is consistent with the fact that ENNs co-occur and share a common biosynthetic enzyme (ESYN1). Their positive correlation with BEA is also supported given that BEA and ENNs are closely related cyclic hexapeptides that are often co-produced due to their shared metabolic pathways (Stepień and Waśkiewicz, 2013; Liuzzi et al., 2017; Xu et al., 2024). The FB pair was positively correlated due to their linked biosynthesis and regulation by the FUM cluster (Singh et al., 2023). The association of ZEA with DON and ADON is in agreement with the frequent co-occurrence of ZEA and type B trichothecenes (Thapa et al., 2021). For the type A trichothecenes (NEO, DAS, HT-2, and T-2), the correlation among themselves is expected given their shared biosynthetic pathways (Wang et al., 2023). Similarly, DON and ADON, both type B trichothecenes, were also positively associated. The correlation between DON and DAS may reflect interactions among trichothecene biosynthetic pathways.

The negative relationship between BEA and ZEA could be explained by a mutual exclusion pattern due to the relatively small number of ZEA-producing *Fusarium* spp. (some clades from FSAMSC and FIESC) compared with the wider range of BEA-producing species (Munkvold et al., 2021) or may be due to an imbalance in our data abundance rather than biological reasons, as we found no reports linking these mycotoxins. Fusarin C and fusaric acid showed a positive association, which may reflect some shared steps of biosynthesis (given that both are polyketides) or both gene clusters being activated by nitrogen excess (Niehaus et al., 2014). Nonetheless, their negative correlation with BEA is not well documented and could be due to the same reasons as in the case of ZEA.

In this context, the high Spearman’s coefficients (*ρ*) indicate the co-production of toxins within individual strains, whereas negative coefficients reflect mutual exclusion in production. However, these results should be interpreted with caution. These negative correlations occur mainly for toxins that are more frequently produced (e.g., BEA), possibly because only those with sufficient variability across strains can generate a detectable inverse pattern, while toxins that are rarely produced (e.g., ZEA) do not contribute enough information for a significant correlation.

### 4.3 Mycotoxin profile driven by intrinsic factors

With regard to the whole toxigenic profile, we visualized the samples in the PCoA space (**Figure 3**) and observed a slight ordination of the strains, consistent with the already discussed production expected for each SC.

The two-group clustering (**Supplementary Table S2**) was consistent with the BEA–ENNs/fusaric acid gradient observed along PCoA1; however, species assignation to one or the other cluster was driven by the less represented species rather than by the dominant ones. As the species identity and SC were significantly different among clusters (Fisher’s exact test: *p*_-adj_ = 0.0075), we interpret these groups as a reflection of the intraspecific variation in production more than discrete, taxonomically defined chemotypes.

When analyzing the variables influencing the whole toxigenic profile, we found species and SC as the only significant ones (**Table 4**), although a significant dispersion (betadisper: *p* = 0.002) indicates that these differences reflect both profile composition and heterogeneity of dispersion, the latter partly related to the unbalanced and small sample sizes of some complexes (Warton et al., 2012). When analyzing the other variables (i.e., soil OM, pH, C/N ratio, and associated crop) using a conditioned tb-RDA (**Supplementary Table S3**), we found that they did not have statistical significance and had a negligible effect (*p* > 0.05, *R*^2-adj^ = 0.006) once the effect of taxonomic identity was accounted for. Although SC explained almost 30% of the variance, the residual variance was 65%, consistent with the intraspecific variability observed in the mycotoxin production of the strains and our inability to measure more edaphic, agronomic, or ecological variables that may be influencing the profiles (Daou et al., 2021; Carro-Huerga et al., 2023; Nikitin et al., 2023; Wang et al., 2024).

We also observed a significant effect of SC over all individual toxins (Kruskal–Wallis: *p*_-adj_ < 0.05) (**Supplementary Table S4**). However, this must be interpreted along with the prevalence to distinguish toxins, in which SC explained a high proportion of the variance due to no other complex producing the toxin (in particular NEO and DAS, with *η*^2^ > 0.75, but with 9.1% of the strains producing them), from the central mycotoxins (BEA and ENNs) that display high effect size and high prevalence, then representing the main differentiators between SCs across the collection.

Given the crucial role of species identity in mycotoxin production, more exhaustive sampling and isolation, designed as a formal abundance study (selecting different species occurring in the soil samples), together with genetic studies on the strains, should be performed to uncover molecular traits that could be shaping the different chemotypes. Whole mycobiome analysis would be remarkably interesting, as the community probably shapes the production profiles of *Fusarium* and other toxigenic fungi (Xu et al., 2020; Todorović et al., 2023), and linking the *Fusarium* spp. chemotypes to agronomical and environmental variables could help in the prediction and management of mycotoxin contamination.

The extensive presence of BEA and the high concentrations of ENNs and BEA found in this study highlight the need for further research on the mycotoxins in Spain and Southern Europe, as we found a strong potential for emerging mycotoxin production in agricultural contexts together with the presence of newly described *Fusarium* spp. that could be phytopathogenic. Nevertheless, direct measurement of the mycotoxins in soil and their effect on plant health should be characterized.

## Data Availability

The original contributions presented in the study are included in the article/**Supplementary Material**. Further inquiries can be directed to the corresponding author.
