# Supplementary material for: Agricultural soils as a potential reservoir of mycotoxigenic Fusarium spp. in northwest Spain
Source: Front Fungal Biol. 2026 Oct 6;7:1946828. doi: 10.3389/ffunb.2026.1946828 (PMC13680485; doi:10.3389/ffunb.2026.1946828)
Supplement: Supplementary file 1 [file DataSheet1.pdf]

Supplementary Material

SM Table S1. Original dataset in .xlsx format. The dataset contains all the information about the 122 total strains, 115 of them used in mycotoxin production analysis: codes, sampling date, location, current and previous associated crop, soil physicochemical properties, species, species complex and mycotoxin production (in µg/kg of rice, being >LOQ values computed as LOQ/2).

File: SM_table_S1_dataset.xlsx

SM Table S2. Exploratory cluster analysis of toxigenic profiles. K-means clustering was performed on the Hellinger-transformed mycotoxin data (N = 107) and reported in an excel file with several sheets. **A)** "Cluster stability" reports cluster number calculated by k = 2-8, Jaccard's index for cluster stability (100 replicates) and stability summarized as "stable" (> 0.85) or "doubtful" (0.60 - 0.85) and "unstable" (< 0.60). **B)** "Cluster size" summarizes the number of strains per cluster and the median of the median number of toxins produced by each strain in the cluster, to reveal the most common number of toxins produced by the "typical" strain of the cluster. **C)** "Cluster profile" reports the median concentration (µg/kg) of each toxin produced in the cluster. **D)** "Cluster association" summarizes the significance (P- value and P-adj with BH correction < 0.01 (**)) of the association tests (Fisher's exact test with Monte Carlo simulation or Wilcoxon rank-sum test) performed between cluster assignation and a set of independent variables. **E** and **F)** report the species identity ("Cluster species") or species complex ("Cluster SC") of the strains in each cluster.

File: SM_table_S2_clustering.xlsx

SM Table S3. Transformation-based redundancy analysis (tb-RDA) of the toxigenic profile conditioned on species complex (SC). This analysis was performed on the Hellinger transformed mycotoxin data (N = 107) with soil parameters (OM, pH, C/N ratio) and associated crop as

constraining variables and SC as conditioning variable. **A)** “Variance partitioning” shows the Inertia (total variance of the Hellinger-transformed data) and the proportion explained by the conditioning variable (SC), constrained variables (soil and crop) and the unexplained residual. **B)** “Marginal test” summarizes the result for the permutation test on each constraining variable (ANOVA by margin, 999 permutations), reporting degrees of freedom (“Df”), variance, pseudo-F statistic (“F”), and permutational P-value (“Pr(>F)”). **C)** “R^2-adj” is the proportion of variance explained by the constrained variables corrected by the number of predictors (R^2-adj = 0.006).

File: SM_table_S3_tbRDA.xlsx

SM Table S4: Differences in individual mycotoxin production among species complexes (SC). Kruskal-Wallis’s test with post-hoc pairwise Dunn’s comparisons on the non-transformed data (N = 110). “n producers” and “% producers” indicate the number and percentage of producer strains (for contextualization), “χ^2” chi-squared statistic; “df” degrees of freedom; “P”, raw P-value; “P-adj”, Benjamini–Hochberg adjusted P-value; “η^2”, eta-squared effect size by which data is ordered (magnitude: large/moderate/small); “Dunn”, compact letter display of Dunn's post-hoc test (BH-adjusted), where different letters indicate significant differences; “Significance” summarizes P-adj < 0.001 (***); P-adj < 0.01 (**), P-adj < 0.05 (*). SC with fewer than 3 isolates were excluded from post-hoc comparisons (FBURSC). Fusarin C is reported on a relative scale. Abbreviations: NEO: neosolaniol; DAS: diacetoxyscirpenol; BEA: beauvericin; ZEA: zearalenone; HT-2: HT-2 toxin; ENN: enniatins B and B1; FB2: fumonisin B2; ENN: enniatins A1 and A; FB1: fumonisin B1; DON: deoxynivalenol.

File: SM_table_S4_KW.xlsx
